# Salvage Resection Following Reassessment of Resectability in Advanced Hepatocellular Carcinoma

**DOI:** 10.7759/cureus.103716

**Published:** 2026-02-16

**Authors:** Dimitrios K Vlachos, Michail Konstantinidis, Dionysios Prevezanos, Nikolaos Machairas, Georgios C Sotiropoulos

**Affiliations:** 1 Department of Liver Transplantation and Hepatobiliary Surgery, Laiko General Hospital of Athens, National and Kapodistrian University of Athens, Athens, GRC

**Keywords:** advanced hcc, bclc staging, hepatectomy, hepatic vein tumor thrombus, inferior vena cava tumor thrombus, portal vein embolization, salvage surgery, surgical resectability

## Abstract

Hepatocellular carcinoma (HCC) management is commonly guided by the Barcelona Clinic Liver Cancer (BCLC) staging system; however, surgical resectability cannot always be accurately defined by staging alone. We report a case highlighting how initial misclassification of resectability led to loss of an early curative opportunity, while subsequent expert reassessment enabled successful salvage surgery.

A 67-year-old man with chronic hepatitis B virus (HBV) infection was diagnosed with a large solitary HCC confined to the right hepatic lobe. Liver function was preserved, and there was no radiological evidence of vascular invasion or extrahepatic disease at presentation. Despite these favorable features, upfront surgical resection was not pursued. The patient underwent right portal vein embolization (PVE), which resulted in adequate hypertrophy of the future liver remnant, confirming technical resectability. Nevertheless, surgery was deferred, and transarterial radioembolization (TARE) was performed. During this interval, tumor progression occurred, with the development of a tumor thrombus in the right hepatic vein extending into the inferior vena cava (IVC), meeting criteria for advanced-stage disease.

Following referral to a specialized hepatobiliary center, multidisciplinary reassessment determined that curative surgery remained feasible despite macrovascular involvement. The patient underwent right hepatectomy with resection of the right hepatic vein and inferior vena cava thrombectomy. Complete tumor removal with negative margins was achieved. The postoperative course was uneventful, and follow-up imaging at six months demonstrated no evidence of recurrence.

This case illustrates the limitations of rigid guideline-based decision-making in HCC and underscores the importance of individualized surgical evaluation in experienced hepatobiliary centers. Continuous reassessment of resectability, particularly after locoregional therapies, may reveal curative options even in patients classified as having advanced disease.

## Introduction

Liver cancer, predominantly hepatocellular carcinoma (HCC), remains one of the most difficult cancers to manage, ranking sixth in global incidence and third in cancer-related mortality [1]. Currently, hepatitis B virus (HBV) infection is the leading cause of HCC, responsible for nearly half of all cases [2]. However, the increasing availability of antiviral therapies and HBV vaccination programs has contributed to a reduction in HBV- and HCV-related liver disease in many regions [2]. In contrast, non-alcoholic steatohepatitis (NASH), associated with metabolic syndrome and diabetes, is emerging as a growing contributor to HCC development [2]. Management of HCC requires an individualized understanding of tumor biology, liver function, and patient performance status. The Barcelona Clinic Liver Cancer (BCLC) staging system is the most widely used algorithm to guide treatment decisions, stratifying patients into stages that correspond to evidence-based therapeutic options [3]. According to BCLC, patients with vascular invasion, including portal vein or hepatic vein thrombus, are classified as advanced-stage (BCLC C) and are typically offered systemic therapy such as tyrosine kinase inhibitors or immunotherapy [3].

While the BCLC system has standardized care globally, it is not without limitations. It may be too rigid in certain real-world clinical scenarios, particularly in centers with advanced hepatobiliary surgical expertise. In carefully selected patients with preserved liver function, good performance status, and anatomically favorable tumors, surgical resection may offer meaningful long-term survival, even in the presence of vascular invasion [4].

This case highlights a patient with a resectable right-lobe HCC who was initially considered not suitable for upfront surgical resection at another institution. The delay in surgical treatment led to tumor progression with right hepatic vein and inferior vena cava (IVC) thrombus. Despite this, the patient underwent a successful right hepatectomy and thrombectomy and remains recurrence-free at six months of follow-up. This case emphasizes the importance of continuous reassessment and individualized, multidisciplinary care in HCC management.

## Case presentation

A 67-year-old Caucasian male patient with no prior history of liver disease presented with a newly diagnosed mass in the right hepatic lobe. He was in excellent clinical condition, and laboratory workup revealed chronic hepatitis B virus infection. Imaging (magnetic resonance imaging (MRI) and computed tomography (CT)) showed a large, solitary mass confined to the right lobe, with no evidence of vascular invasion or extrahepatic spread. Liver function was preserved (Child-Pugh A, Model for End-Stage Liver Disease (MELD) 8), and the left lobe appeared healthy.

Despite this, the patient was evaluated at another center, where the tumor was deemed borderline resectable. The surgical team recommended right portal vein embolization (PVE) to induce hypertrophy of the left lobe. PVE was successfully completed, and follow-up imaging confirmed adequate hypertrophy of the future liver remnant. Despite this, the tumor was subsequently considered inoperable at the referring institution; the specific rationale for this decision was not clearly documented in the available records. Transarterial radioembolization (TARE) was therefore proposed as a palliative treatment.

The patient underwent TARE using Yttrium-90 microspheres. Three months later, follow-up imaging revealed a good partial response of the tumor; however, a new tumor thrombus was noted in the right hepatic vein, extending into the inferior vena cava (IVC). The patient was referred to our hepatobiliary surgical unit at this stage.

**Figure 1:**
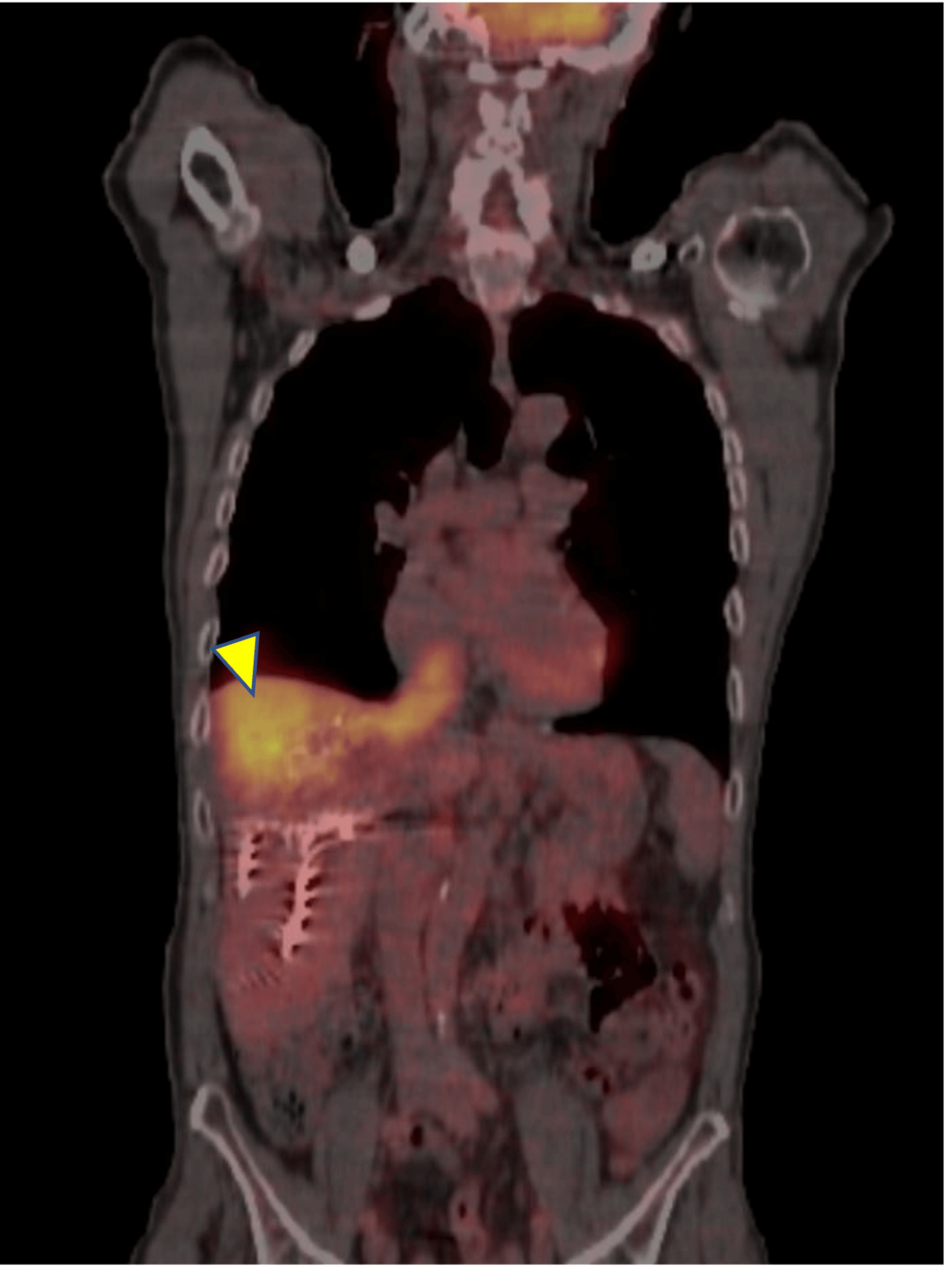
18F-FDG PET/CT imaging Coronal fused 18F-FDG PET/CT image demonstrating increased tracer uptake corresponding to a tumor thrombus extending from the right hepatic vein into the inferior vena cava (arrowhead). 18F-FDG PET/CT: fluorine-18 fluorodeoxyglucose positron emission tomography/computed tomography

**Figure 2:**
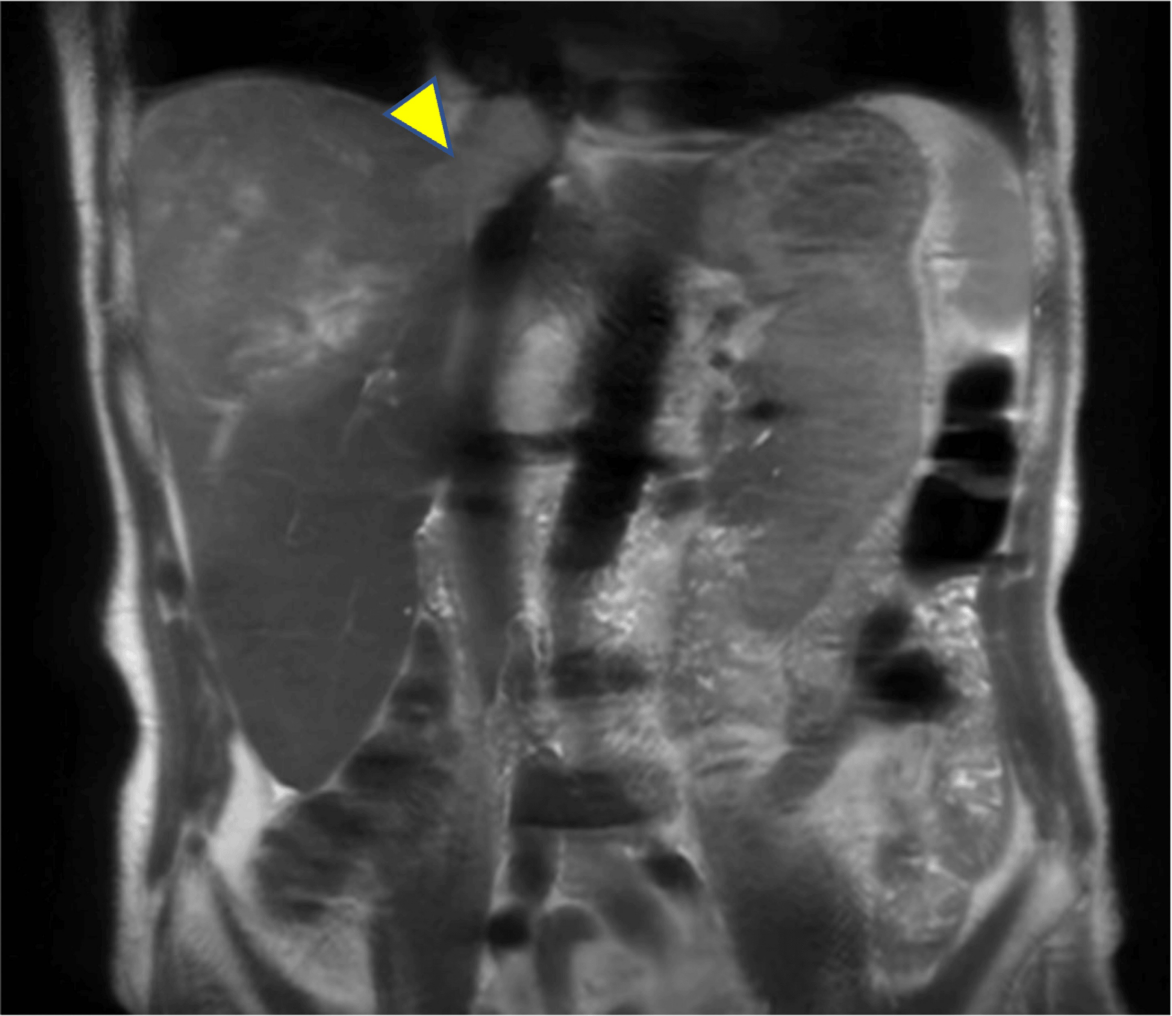
Contrast-enhanced magnetic resonance imaging (coronal plane) Coronal contrast-enhanced magnetic resonance imaging demonstrating an intraluminal tumor thrombus arising from the right hepatic vein and extending into the inferior vena cava (arrowhead), without radiological evidence of caval wall invasion.

**Figure 3:**
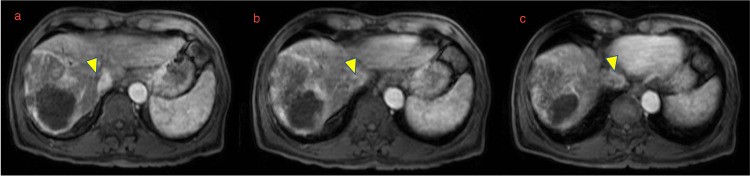
Sequential axial contrast-enhanced magnetic resonance imaging demonstrating tumor thrombus progression Sequential axial contrast-enhanced magnetic resonance imaging demonstrating (a) tumor thrombus within the right hepatic vein, (b) cranial progression of the thrombus toward the hepatocaval confluence, and (c) extension of the thrombus into the inferior vena cava. Arrowheads indicate the tumor thrombus.

Upon referral to our center, the patient underwent further staging with fluorine-18 fluorodeoxyglucose positron emission tomography/computed tomography (18F-FDG PET/CT) (Figure 1) and contrast-enhanced MRI (Figure 2 and Figure 3). These investigations confirmed the partial response of the primary hepatic lesion and demonstrated the presence and extent of a tumor thrombus originating from the right hepatic vein and extending into the inferior vena cava, without evidence of extrahepatic disease or distant metastases. 

Based on this comprehensive reevaluation, we considered curative resection to be feasible at the time of our assessment. Despite the development of a tumor thrombus extending into the right hepatic vein and inferior vena cava (IVC), we considered surgery still feasible. The patient had preserved liver function and no evidence of metastatic disease, and remained in excellent overall condition, all of which supported proceeding with a right hepatectomy and thrombectomy.

Preoperative planning focused on the extent of the thrombus and its relationship to the IVC wall. Imaging suggested that the thrombus was intraluminal and not invading the vessel wall of the IVC. Nevertheless, due to the potential complexity of the dissection and risk of intraoperative complications, the procedure was planned with enhanced safety measures. A cardiothoracic surgeon was present during the operation, and a cardiopulmonary bypass system was prepared and on standby in the event vascular control of the IVC or cardiac support became necessary.

During surgery, a tumor thrombus extending from the right hepatic vein into the IVC was identified. The thrombus was carefully extracted through a controlled venotomy at the right hepatic vein, without the need for IVC wall resection, as it was non-adherent and entirely intraluminal. Following successful thrombectomy, a right hepatectomy was performed along with en bloc resection of the right hepatic vein. The procedure was completed under vascular control, and all margins were histologically confirmed negative (R0 resection). The total operative time was 280 minutes, with an estimated blood loss of 500 mL. No intraoperative blood transfusions were required.

The patient had an uneventful postoperative course and was discharged in good condition on postoperative day 10. Histopathological examination demonstrated a 10-cm hepatocellular carcinoma with macrotrabecular growth pattern and moderate to poor differentiation (WHO grade 3). Multiple vascular tumor emboli were identified, including invasion of a major hepatic venous branch. Areas of treatment-related necrosis accounted for approximately 30% of the tumor volume, consistent with prior transarterial radioembolization. Surgical margins were negative (R0 resection). The surrounding liver parenchyma showed chronic hepatitis B-related changes with advanced fibrosis. Follow-up imaging at six months showed no evidence of disease recurrence.

## Discussion

This case highlights the evolving role of surgical resection in HCC with macrovascular invasion, challenging the traditional notion that such patients are universally unresectable. Although BCLC stage C typically directs treatment toward systemic therapy, growing evidence supports surgery in select cases with preserved liver function, good performance status, and technically resectable tumors.

Multiple comparative studies have demonstrated the survival advantage of surgical resection in patients with vascular involvement. For example, Wang et al. reported a median survival of 19 months for patients undergoing surgery for inferior vena cava tumor thrombosis (IVCTT) or right atrial thrombus, compared to 4.5 months with TACE and five months with supportive care​ [5]. Similarly, Chen et al. showed a median survival of 27.1 months in patients with type I hepatic vein tumor thrombus (HVTT) treated surgically, with outcomes decreasing in more advanced thrombus classifications, but still superior to non-surgical options​ [4]. A meta-analysis comparing surgery to external beam radiotherapy (EBRT) in IVC/right atrium (RA) thrombus cases found that one-year survival was significantly higher with surgery (62.4% versus 48.8%), and median survival was also longer (15.3 months versus 11.7 months)​ [6]. Additional support comes from Wakayama et al., who found a median survival of 30.8 months after curative resection for HCC with vascular invasion, significantly better than historical outcomes with sorafenib (typically under 10 months) [7].

Sakamoto and Nagano reported that surgical resection in patients with HCC with IVC tumor thrombus can result in a median survival of 19-30.8 months, especially in tumors under 10 cm or when a partial response to prior locoregional therapy is observed​. These findings support the role of surgery even in advanced cases with vascular involvement, provided that patients are carefully selected and treated in centers with the required expertise [8].

Our patient had type II IVCTT, which is generally considered surgically manageable in the absence of portal vein invasion. The classification by Chen et al. stratifies tumor thrombi into types I-III, with gradually worsening prognosis, but still highlights surgery as viable in types I and II when performed in specialized centers [9].

Our patient was initially considered borderline resectable and underwent portal vein embolization (PVE) followed by radioembolization, but no surgical attempt was made despite adequate hypertrophy. During this period, the tumor progressed to involve the hepatic vein and IVC. This outcome underscores a critical clinical point: continuous multidisciplinary reassessment is essential following PVE, radioembolization, or any locoregional therapy. In our center, a second evaluation recognized that resection was still feasible despite vascular involvement. Surgical resectability in HCC should be regarded as a dynamic process rather than a static decision based solely on initial staging.

While systemic therapies and immunotherapy-based combinations continue to evolve, their median survival benefit in patients with macrovascular invasion remains limited. For instance, the SARAH trial found no significant difference in survival between radioembolization and sorafenib, with both arms reporting median survivals under 10 months​ [10]. Novel regimens, such as atezolizumab-bevacizumab, offer improved outcomes but are not yet considered curative in most cases [2].

## Conclusions

In conclusion, our case reinforces that surgical resection should remain a strong consideration for select patients with HCC and vascular invasion, even beyond current guideline-based restrictions. Reevaluation after initial therapy, especially at centers with hepatobiliary expertise, can uncover potentially curative options that were not initially pursued. Clinical staging systems are valuable, but flexibility and multidisciplinary judgment are essential to optimizing outcomes in complex HCC cases.
